# Structural Racism and HIV Pre-exposure Prophylaxis Use in the Nationwide US: A County-Level Analysis

**DOI:** 10.1007/s40615-024-02127-5

**Published:** 2024-08-13

**Authors:** Fanghui Shi, Tianyue Mi, Xiaoming Li, Huan Ning, Zhenlong Li, Xueying Yang

**Affiliations:** 1 Arnold School of Public Health, South Carolina SmartState Center for Healthcare Quality, University of South Carolina, Columbia, SC 29208 USA; 2 Department of Health Promotion, Education and Behavior, Arnold School of Public Health, University of South Carolina, 915 Greene Street, Columbia, SC 29208 USA; 3 Geoinformation and Big Data Research Laboratory, Department of Geography, The Pennsylvania State University, University Park, PA 16802 USA

**Keywords:** Structural racism, Pre-exposure prophylaxis, HIV, County level

## Abstract

**Background:**

Structural racism contributes to geographical inequalities in pre-exposure prophylaxis (PrEP) coverage in the United States (US). This study aims to investigate county-level variability in PrEP utilization across diverse dimensions of structural racism.

**Methods:**

The 2013–2021 nationwide county-level PrEP rate and PrEP-to-need ratio (PNR) data were retrieved from AIDSVu. PrEP rate was defined as the number of PrEP users per 100,000 population, and PNR was defined as the ratio of PrEP users to new HIV diagnoses per calendar year. Linear mixed effect regression was employed to identify associations of county-level structural racism (e.g., structural racism in housing and socioeconomic status) with PrEP rate and PNR on a nationwide scale of the US.

**Results:**

From 2013 to 2021, the mean PrEP rate and PNR increased from 3.62 to 71.10 and from 0.39 to 10.20, respectively. Counties with more structural racism in housing were more likely to have low PrEP rates (adjusted *β* =  − 5.80, 95% CI [− 8.84, − 2.75]). Higher PNR was found in counties with lower structural racism in socioeconomic status (adjusted *β* =  − 2.64, 95% CI [− 3.68, − 1.61]). Regionally, compared to the Midwest region, counties in the West region were more likely to have higher PrEP rate (adjusted *β* = 30.99, 95% CI [22.19, 39.80]), and counties in the South had lower PNR (adjusted *β* =  − 1.87, 95% CI [− 2.57, − 1.17]).

**Conclusions:**

County-level structural racism plays a crucial role in understanding the challenges of scaling up PrEP coverage. The findings underscore the importance of tailored strategies across different regions and provide valuable insights for future interventions to optimize PrEP implementation.

**Supplementary Information:**

The online version contains supplementary material available at 10.1007/s40615-024-02127-5.

## Introduction

Pre-exposure prophylaxis (PrEP) is a medicine taken in the form of pills or shots that reduces the chance of contracting HIV from injection drug use or sex. Throughout the last decade, PrEP has emerged as a cornerstone in HIV prevention across the US [1]. Demonstrating remarkable efficacy, PrEP reduces the risk of HIV transmission through sexual contact by 99% and through injection drug use by 74% [2]. In response to the ongoing HIV epidemic, the US Department of Health and Human Services launched the Ending the HIV Epidemic in the US (EHE) initiative in 2019. This initiative aims to reduce new HIV infections by 90% by 2030, with a key objective of expanding PrEP utilization to cover at least 50% of the 1.2 million individuals estimated to be at risk of HIV acquisition in the US [1]. Despite a gradual increase in PrEP adoption nationwide, recent estimates suggest that only 30% of the estimated 1.2 million people who could benefit from PrEP were prescribed PrEP medications in 2021 [3]. Additional strategies and resources are needed to understand obstacles to PrEP use and address unmet needs to increase PrEP coverage.

Persistent racial inequities in PrEP use are evident. According to CDC data from 2022, Black individuals represented 42% of new HIV diagnoses but accounted for only 14% of PrEP users [4]. Nationwide surveillance data reveal a stark contrast in PrEP coverage, with rates at 8.0% for Black individuals and 61.1% for White individuals [5]. At the individual level, limited awareness of PrEP, insufficient knowledge about PrEP’s benefits, and competing priorities for food, shelter, employment, and other health needs impede Black individuals from seeking and adhering to PrEP [6–8]. At the community level, clinicians’ concerns about risk compensation and the worry that PrEP use will increase risk behaviors decrease PrEP prescription toward Black individuals due to stereotypes suggesting greater sexual risk-taking among Black individuals [9, 10]. At the societal level, a lower density of PrEP-providing clinics in Black communities and transportation challenges exacerbate the difficulties Black individuals face in accessing PrEP services [11, 12].

Structural racism could also be a salient population-level determinant of low PrEP coverage, but this has been understudied in the US [13–16]. Reporting racial disparities in the coverage of PrEP without taking structural racism into account would reinforce unspoken yet persistent stereotypes of racial minority groups, which undermines efforts to eliminate health inequalities. Among studies on the population-level disparities in health, structural racism is usually described as the “fundamental cause” [17, 18]. Structural racism refers to the totality of how societies foster racial discrimination through multiple pathways (e.g., education, employment, healthcare, housing, and criminal justice) [17]. Structural drivers place racial minority groups at greater risk for HIV and impair their access to HIV prevention tools, including PrEP. A county-level analysis using person-level prescription data by combing records from pharmacies, clinics, and payers found that each 5% increase in proportion of Black residents among counties was associated with a 5% decrease in PrEP-to-need ratio (PNR), defined as the ratio of PrEP users to new HIV diagnoses [19]. However, this study only explored one dimension of structural racism, structural racism in housing, and used the percentage of Black residents as a proxy measure. Additionally, most of existing studies that described the relationship between structural racism and insufficient PrEP use were based on qualitative methods only [20, 21]. There is a need for studies that quantify the measurement of multiple dimensions of structural racism to provide empirical evidence for the observed relationship.

Gaining insights into the impacts of structural racism from a population perspective could provide evidence for future policymaking endeavors and potential structural-level strategies to enhance PrEP promotion beyond individual-level interventions [22]. This study aims to explore the effect of multiple quantified indicators of structural racism on the statewide county-level variations of PrEP rate and PrEP-to-need ratio (PNR) from 2013 to 2021. By comprehending the fluctuation in county-level PrEP utilization across diverse dimensions of structural racism, it becomes feasible to prioritize county-level initiatives to enhance PrEP uptake in the US.

## Methods

### Data Sources and Retrieval

Nationwide County-level PrEP rate and PNR data from 2013 to 2021 were retrieved from AIDSVu.org, which compiled all HIV surveillance data by the Centers for Disease Control and Prevention (CDC). Although available, PrEP rate and PNR data for 2022 and 2023 were not included in the current study because, at the time of analysis, key main county-level characteristics of interest, including three house-related structural racism variables, were only available up to 2021. According to AIDSVu’s PrEP/PNR data methods, the yearly PrEP rate was calculated as the yearly number of PrEP users per 100,000 population. The yearly number of PrEP users was defined as the number of people prescribed TDF/FTC or TAF/FTC for PrEP in a calendar year. PrEP rate is a traditional metric indicating the overall PrEP prevalence [19]. PNR was calculated as the yearly number of PrEP users divided by the number of persons newly diagnosed with HIV at each county during the calendar year. PNR was used to describe the distribution of PrEP prescriptions in relation to the epidemic need and is suggested to reflect the sufficiency of PrEP use at the area level. Other county-level factors were extracted from the 5-year estimate American Community Survey (ACS). The unique Federal Information Processing Standards (FIPS) code for each county was used to link the two datasets utilized in the current study.

### Structural Racism

Structural racism was measured by racial disparities in housing and socioeconomic status [23]. Structural racism in housing was assessed by spatial proximity index, Black/White dissimilarity index, and delta. The spatial proximity index is a measure of clustering, and it measures the extent to which neighborhoods inhabited by minorities adjoin one another. The spatial proximity is larger than one when members of the minority group live nearer to one another than to members of the other group and is smaller than one otherwise. The Black/White dissimilarity index is a measure of evenness, and it indicates the degree to which White and Black county residents live separately from each other [24]. The dissimilarity index score can be interpreted as the percentage of one racial resident that has to move to a different geographic area to produce a distribution that of the larger area. Thus, the higher the score of the residential segregation index, the greater the residential segregation between two racial groups. Delta is a measure of concentration, and it indicates the relative amount of physical space occupied by the minority group. This index can be interpreted as the proportion of the minority group that has to move across census tract to achieve uniform density. It ranges from 0 to 1, with a larger value indicating high segregation. Detailed information regarding the formula for all the index calculations of structural racism in housing is described in supplemental materials. Structural racism in socioeconomic status was calculated by the ratio of the proportion of unemployed Black residents aged ≥ 16 to the proportion of unemployed White residents aged ≥ 16. Age 16 was used as the cutpoint for the unemployment proportion because the US labor force is restricted to people at least 16 years old. All the needed data for calculation were extracted from ACS 5-year estimates.

### County-Level Model Covariates

Five county-level basic demographic- and healthcare resource–related factors were extracted from ACS and adjusted as potential confounders in the analysis, including the percentage of non-Hispanic Black, the percentage of the population who were older than 65 years old, the number of violent crimes per 100,000, the percentage of population with no health insurance coverage, and population density. In addition, counties were categorized into four regions based on the US Census groupings of states by regions, including Midwest, Northeast, South, and West regions.

### Statistical Analysis

The distribution of county-level characteristics overall and by year was described using three quantiles (25th percentile, 50th percentile, and 75th percentile). The temporal trends of county-level PrEP rate and PNR by four US regions from 2013 to 2021 were illustrated by two locally weighted scatterplot (LOESS) smoothing curves generated by “ggplot2” package in R software. To explore the association of multiple measurements of structural racism with PrEP rate and PNR, linear mixed effects models with FIPS code (the unique identifier for each county) added as a random effect were conducted. In the crude model for both PrEP rate and PNR, structural racism–related variables were put in different models separately, and the calendar year and the region were put as additional covariates. In the adjusted model, all the county-level variables, including structural racism–related variables and other county-level covariates were put in the model together, adjusting for calendar year and the region. We standardized all county-level characteristics to deal with varied scales of these characteristics and make models more stable. All analyses were conducted in R version 4.1.2, and *p*-value less than 0.05 was treated as statistically significant level.

## Results

### Descriptive Statistics

**Fig. 1:**
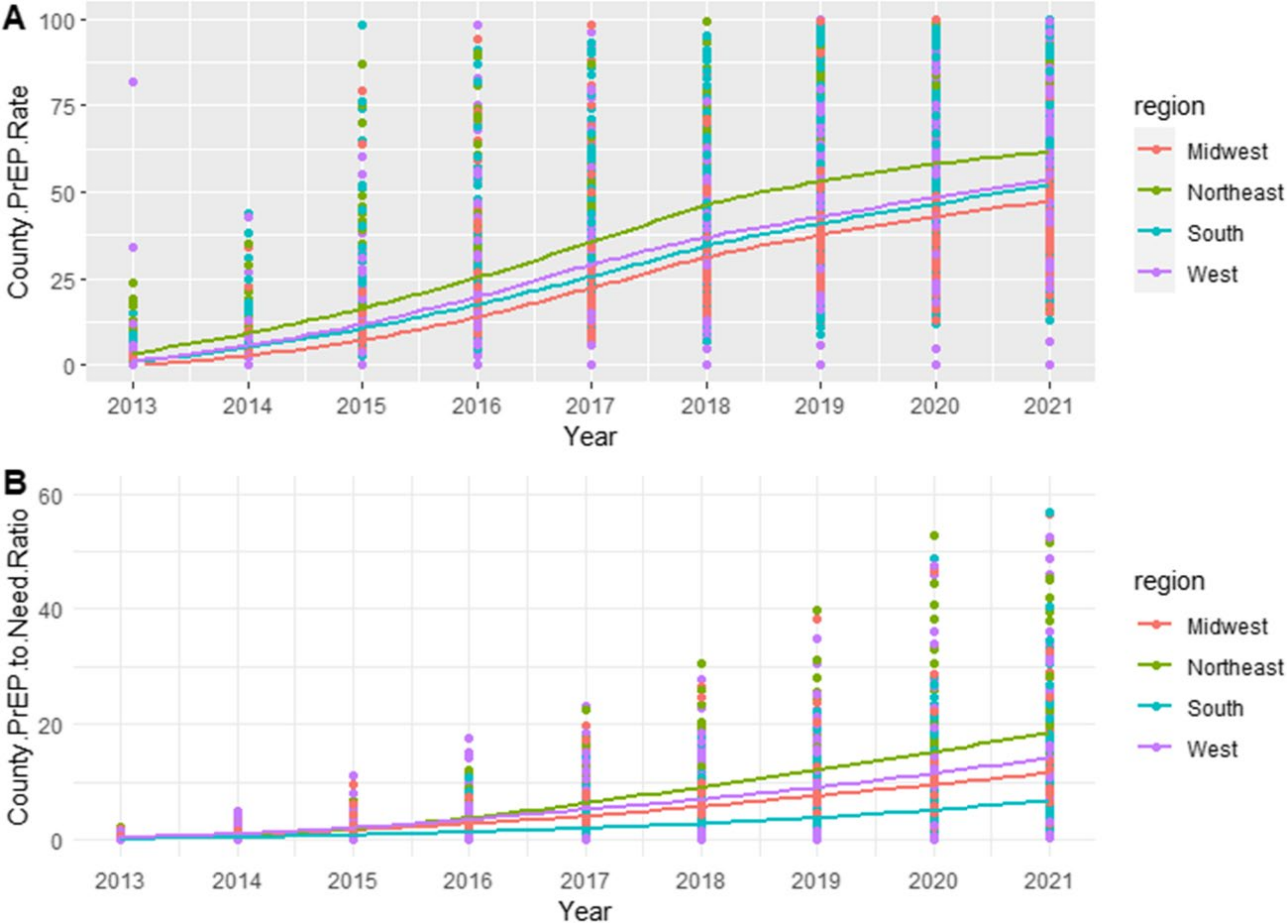
LOESS smooth curve of PrEP rate and PrEP-to-need ratio by four regions in the United States from 2013 to 2021

From 2013 to 2021, there was an overall increasing trend for county-level PrEP coverage, with the average PrEP rate and PNR increasing from 3.62 to 71.10 and from 0.39 to 10.20, respectively. Counties in the Northeast region consistently had the highest PrEP rate across years, followed by West, South, and Midwest regions. Regarding PNR, the Northeast region also had the highest PNR across years, followed by the West, Midwest, and South regions (Fig. 1). County-level spatial proximity index across years was relatively stable, with the quantile being 1.01 [1.00, 1.06] in most of the years. The median values for dissimilarity index and delta were 0.4 and 0.58, indicating around half of the Black residents need to move across census tract to achieve uniform density. The median ratio of Black to White unemployed was 1.61, with the 25th percentile and 75th percentile being 0.28 and 2.56 (Table 1).

**Table 1:** Descriptive distribution of county-level characteristics in the United States over years

	Overall Median [25th, 75th]	Calendar year		
		2013 Median [25th, 75th]	2017 Median [25th, 75th]	2021 Median [25th, 75th]
Spatial proximity index	1.01 [1.00, 1.06]	1.01 [1.00, 1.06]	1.01 [1.00, 1.06]	1.01 [1.00, 1.06]
Dissimilarity index	0.400 [0.26, 0.51]	0.424 [0.29, 0.53]	0.414 [0.28, 0.52]	0.333 [0.20, 0.46]
Delta	0.575 [0.36, 0.74]	0.603 [0.38, 0.76]	0.589 [0.38, 0.75]	0.488 [0.30, 0.66]
BW_unemployed	1.61 [0.28, 2.56]	1.70 [0.71, 2.52]	1.62 [0, 282]	1.47 [0.00, 2.48]
non-Hispanic Black%	0.02 [0.01, 0.10]	0.0212 [0.01, 0.10]	0.0221 [0.01, 0.10]	0.0223 [0.01, 0.10]
Violent crime	202.6 [114.8, 334.6]	211 [116.5, 357.8]	198 [112.7, 325.4]	205 [117.6, 335.0]
Uninsured%	0.17 [0.12, 0.23]	0.220 [0.18, 0.27]	0.166 [0.12, 0.21]	0.125 [0.09, 0.17]
≥ 65 years old%	0.18 [0.15, 0.20]	0.158 [0.13, 0.18]	0.177 [0.15, 0.20]	0.189 [0.16, 0.22]
Population density	46.39 [17.34, 132.74]	46.6 [17.56, 129.85]	46.3 [17.29, 131.88]	46.3 [17.01, 135.76]

*BW_unemployed* the Black to White ratio of unemployment rate, *Violent crime* the number of violent crimes per 100,000 residents

### Linear Mixed Effects Regression Models

Regarding structural racism-related factors, higher dissimilarity index (crude *β* =  − 3.68, 95% CI [− 5.75, − 5.70]), delta (crude *β* =  − 3.50, 95% CI [− 5.70, − 1.30]), and Black to White unemployment rate (crude *β* =  − 3.85, 95% CI [− 6.42, − 1.27]) were related to lower PrEP rate in the crude model, and the significant relationship remained only for dissimilarity index (adjusted *β* =  − 5.80, 95% CI [− 8.84, − 2.75]) in the adjusted model. As for PNR, higher spatial proximity index (crude *β* =  − 0.12, 95% CI [− 0.50, − 0.02]) and delta index (crude *β* =  − 0.16, 95% CI [− 0.64, − 0.00]) were associated with lower PNR in the crude models. In addition, counties with higher Black to White unemployment rates tend to have lower PNR in the adjusted model (adjusted *β* =  − 1.03, 95% CI [− 1.32, − 0.75]).

For other county-level covariates, counties with higher percentages of uninsured adults (adjusted *β* =  − 4.30, 95% CI [− 6.78, − 1.81]) and higher percentages of population older than 65 years old (adjusted *β* =  − 3.90, 95% CI [− 6.72, − 1.07]) were more likely to have low PrEP rate. Regarding PNR, higher PNR was found in counties with lower percentages of non-Hispanic Black (adjusted *β* =  − 1.03, 95% CI [− 1.32, − 0.75]) and lower percentage of population older than 65 years old (adjusted *β* =  − 0.30, 95% CI [− 0.54, − 0.05]). Regionally, compared to counties in the Midwest region, counties in the West region were significantly more likely to have higher PrEP rate (adjusted *β* = 30.99, 95% CI [22.19, 39.80]) and higher PNR (adjusted *β* = 1.02, 95% CI [0.23, 1.82]). Moreover, counties in the South had lower PNR (adjusted *β* =  − 1.87, 95% CI [− 2.57, − 1.17]), and counties in the Northeast (adjusted *β* = 2.27, 95% CI [1.48, 3.06]) had higher PNR (Table 2).

**Table 2:** The association of county-level variables with PrEP rate and PrEP-to-need ratio in the United States from 2013 to 2021

	PrEP rate		PNR	
	Crude beta (95% CI)^a^	Adjusted beta (95% CI)^b^	Crude beta (95% CI)^a^	Adjusted beta (95% CI)^b^
Spatial proximity index	0.883 (− 1.557, 3.322)	0.380 (− 1.876, 2.645)	0.121 (− 0.496, − 0.0211208)*	− 0.011 (− 0.277, 0.256)
Dissimilarity index	− 3.680 (− 5.746, − 1.614)***	− 5.799 (− 8.842, − 2.745)***	0.148 (− 0.494, 0.085)	0.203 (− 0.233, 0.638)
Delta	− 3.500 (− 5.697, − 1.303)**	0.865 (− 2.365, 4.076)	0.163 (− 0.644, − 0.004)*	− 0.734 (− 1.169, − 0.299)
BW_unemployed	− 3.846 (− 6.421, − 1.272)**	− 0.963 (− 3.150, 1.226)	0.148 (− 0.080, 0.499)	− 2.643 (− 3.676, − 1.610)***
Non-Hispanic Black%	~	1.654 (− 1.795, 5.101)	~	− 1.034 (− 1.318, − 0.749)***
Violent crime rate	~	− 1.519 (− 3.978, 0.954)	~	− 0.108 (− 0.326, 0.111)
Uninsured%	~	− 4.302 (− 6.784, − 1.810)***	~	− 0.183 (− 0.441, 0.075)
≥ 65 years old%	~	− 3.896 (− 6.721, − 1.068)**	~	− 0.296 (− 0.542, − 0.050)*
Population density	~	18.922 (16.718, 21.113)***	~	0.422 (0.305, 0.539)***
Year	~	11.224 (10.571, 11.876)***	~	1.518 (1.442, 1.595)***
Region: Northeast versus Midwest	~	6.775 (− 2.971, 16.529)	~	2.271 (1.479, 3.063)***
Region: South versus Midwest	~	4.279 (− 2.882, 11.428)	~	− 1.868 (− 2.565, − 1.171)***
Region: West versus Midwest	~	30.995 (22.188, 39.799)***	~	1.022 (0.229, 1.815)*

^a^Only one structural racism–related factors was put in the model, adjusting for calendar year and the region

^b^All the county-level variables were put in the model together, adjusting for calendar year and the region

*p < 0.05, **p < 0.01, ***p < .001

## Discussion

This study is one of the first attempts to investigate the association between quantified indicators of multiple dimensions of structural racism and PrEP coverage at the county level in the US. Our study provided empirical evidence for the observed relationship between structural racism and insufficient PrEP use observed in existing qualitative studies. We found that structural racism in housing and structural racism in socioeconomic status were associated with lower PrEP rate and lower PNR, respectively, even after controlling for various county-level covariates. Additionally, significant geographic disparities in PrEP were found, with counties in the South region had the lowest PNR in the US, and counties in the West region had high PrEP prevalence.

Structural racism in housing and socioeconomic status were found to be significant barriers to PrEP uptake. Social determinants of health (SDoH) are deeply rooted in structural racism [25, 26] and persistently contribute to disparities in PrEP uptake [18] through downstream variations in community-level socioeconomic mobility, built environments, and access to health services [27]. For example, counties with higher concentrations of Black residents were found to be less likely to have clinics that could prescribe PrEP [15], and this could help explain the association between structural racism in housing and PrEP prevalence found in the current study. Additionally, previous studies have proved that other neighborhood contexts, such as median household income, percent unemployed, and percent uninsured, may influence PrEP coverage [2, 19, 28, 29]. Racial disparities in employment may lead to disparities in poverty and housing instability, which prevent Black communities from adopting PrEP [20]. Costs of paying for medical visits and medications have been frequently cited as barriers to adopting or sustaining PrEP [30]. To address these disparities, well-resourced campaigns and interventions grounded in evidence-based practice are required in counties showing high levels of structural racism.

Significant geographic disparities persist in PrEP utilization across the US [31]. Residents of Southern areas exhibit notably lower PNR compared to those outside the South, attributable to higher concentrations of people of color, lower economic status and mobility, limited access to PrEP-providing clinics, and heightened levels of HIV-related stigma [15, 32, 33]. In 2022, the South accounted for 52% of HIV diagnoses but only represented 38% of PrEP users. For every new HIV diagnosis, there were 22 individuals on PrEP in the Northeast, compared to only ten individuals in the South, highlighting an unmet need for PrEP in Southern regions relative to other areas [4]. Meanwhile, PrEP-prescribing locations were unequally distributed across EHE areas in the southeastern US [34]. The ratio of PrEP-prescribing clinicians per 100 individuals who could benefit from PrEP is notably lower in the South at 4.4 compared to 8.5 in the Northeast [35]. Many large spatial areas, such as South Carolina, Florida, and Georgia, lacked PrEP-prescribing locations and were characterized by high HIV burdens. This creates significant transportation barriers and necessitates individuals to traverse multiple counties to access PrEP services [34]. Multisectoral responses to addressing the structural, policy, and capacity challenges to increase PrEP coverage are needed to achieve equitable PrEP uptake in South areas with high HIV burden.

Some limitations need to be acknowledged for the current study. First, causal relations cannot be assumed because the associations are ecological, and there might be some unmeasured confounding factors. Second, we used publicly available data for analysis, which has the limitation of incomplete data due to data suppression. Third, we were not able to examine the mechanisms of the observed associations based on the current analysis. However, our study still could provide valuable information on the county-level disparities in PrEP and set the foundation for further analysis. Fourth, data on PrEP usage categorized by adult status were unavailable in the AIDSVu database. This limitation prevented us from examining disparities in PrEP use between adults and adolescents under 18 years of age.

## Conclusion

Structural racism is recognized as one of the underlying causes of disparity in PrEP uptake. Failure to recognize and address these barriers would impede the objectives of the EHE initiative and perpetuate racial and ethnic disparities. Further research should consider interventions targeting muti-level dimensions of structural racism to reduce PrEP disparities in historically disadvantaged communities. By prioritizing interventions in counties showing structural racism in housing and socioeconomic status, public health initiatives can work towards ensuring widespread and equitable distribution of HIV prevention efforts across diverse communities.

## Supplementary Information

Below is the link to the electronic supplementary material.

Supplementary file1 (DOCX 19 KB)

## Data Availability

All data used in this study are publicly available and can be accessed through the following sources. 1. AIDSVu: https://aidsvu.org/resources/#/datasets 2. American Community Survey: https://data.census.gov No additional data were analyzed in this study.
