# Supplementary material for: Structural Racism and HIV Pre-exposure Prophylaxis Use in the Nationwide US: A County-Level Analysis
Source: J Racial Ethn Health Disparities. 2024 Aug 13;12(5):3233–9. doi: 10.1007/s40615-024-02127-5 (PMC11822042; doi:10.1007/s40615-024-02127-5)
Supplement: Supplementary file 1 — Supplementary file1 (DOCX 19 KB) [file 40615_2024_2127_MOESM1_ESM.docx]

1. ***Spatial proximity index (SPI)***

$$SPI=\frac{M\times P_{xx}+R\times P_{rr}}{T\times P_{tt}}$$

2. ***Black/White dissimilarity index***

$$ID=0.5\sum_{i=1}^{N} \left| \frac{m_{i}}{M}-\frac{r_{i}}{R} \right|$$

3. ***Delta***

$$Delta=0.5\sum_{i=1}^{N} \left| \frac{m_{i}}{M}-\frac{a_{i}}{A} \right|$$

| **Term** | **Definition** |
| --- | --- |
| N | Number of census tracts, ranked smallest to largest by land area |
| m | Number of census tracts in the county, ranked by increasing distance from the population centroid. |
| $m_{i}$ | Number of the non-Hispanic Black of census tract *i* |
| $M$ | Number of total non-Hispanic Black population |
| $r_{i}$ | Number of the non-Hispanic White population of census tract *i* |
| $R$ | Number of total non-Hispanic White population |
| T | Number of total population |
| $a_{i}$ | The land area of census tract *i* |
| A | The total land area |
| d_ij_ | The distance between area I and area j centroids, where dii = (0.6a_i_)^0.5^ |
| c_ij_ | The exponential transform of -dij [= exp(-d_ij_)] |
